# Hyperandrogenemia in Polycystic Ovary Syndrome: Exploration of the Role of Free Testosterone and Androstenedione in Metabolic Phenotype

**DOI:** 10.1371/journal.pone.0108263

**Published:** 2014-10-13

**Authors:** Elisabeth Lerchbaum, Verena Schwetz, Thomas Rabe, Albrecht Giuliani, Barbara Obermayer-Pietsch

**Affiliations:** 1 Department of Internal Medicine, Division of Endocrinology and Metabolism, Medical University of Graz, Graz, Austria; 2 University Women's Hospital, Heidelberg, Germany; 3 Department of Obstetrics and Gynecology, Medical University of Graz, Graz, Austria; Medical University of Graz, Austria

## Abstract

**Objective:**

To evaluate the association between androstenedione, testosterone, and free testosterone and metabolic disturbances in polycystic ovary syndrome.

**Methods:**

We analyzed the association between androstenedione, testosterone, and free testosterone and metabolic parameters in a cross-sectional study including 706 polycystic ovary syndrome and 140 BMI-matched healthy women. Polycystic ovary syndrome women were categorized into 4 groups: normal androstenedione and normal free testosterone (NA/NFT), elevated androstenedione and normal free testosterone (HA/NFT), normal androstenedione and elevated free testosterone (NA/HFT), elevated androstenedione and free testosterone (HA/HFT).

**Results:**

Polycystic ovary syndrome women with elevated free testosterone levels (HA/HFT and NA/HFT) have an adverse metabolic profile including 2 h glucose, HbA1c, fasting and 2 h insulin, area under the insulin response curve, insulin resistance, insulin sensitivity index (Matsuda), triglycerides, total and high density lipoprotein cholesterol levels compared to NA/NFT (p<0.05 for all age- and BMI-adjusted analyses). In binary logistic regression analysis adjusted for age and BMI, odds ratio for insulin resistance was 2.78 (1.34–5.75, p = 0.006) for polycystic ovary syndrome women with HA/HFT compared to NA/NFT. We found no significantly increased risk of metabolic disorders in polycystic ovary syndrome women with HA/NFT. In multiple linear regression analyses (age- and BMI-adjusted), we found a significant negative association between androstenedione/free testosterone-ratio and area under the insulin response curve, insulin resistance, and total cholesterol/high density lipoprotein cholesterol-ratio and a positive association with Matsuda-index, and high density lipoprotein cholesterol (p<0.05 for all).

**Conclusions:**

Polycystic ovary syndrome women with elevated free testosterone levels but not with isolated androstenedione elevation have an adverse metabolic phenotype. Further, a higher androstenedione/free testosterone-ratio was independently associated with a beneficial metabolic profile.

## Introduction

Polycystic ovary syndrome (PCOS) is the most common endocrine disease in reproductive-aged women and affects up to 20% of all women when using Rotterdam criteria [1]. PCOS women present with hyperandrogenism, oligo- or amenorrhea, and polycystic ovaries (PCO). Further, metabolic disturbances such as central obesity, insulin resistance (IR), a disturbed glucose metabolism, and the metabolic syndrome (MS) are common findings in women with PCOS [2], [3], [4], [5]. There is accumulating evidence that high androgen levels are the fundamental factor in the pathogenesis of PCOS [6]. High testosterone levels are linked with obesity, in particular with an abdominal fat distribution [7], as well as with IR and a higher prevalence of glucose intolerance [6]. Further, hyperandrogenism leads to PCO morphology and ovulatory dysfunction in animal models as well as in women with PCOS [8], [9]. Those androgens are produced in the ovary as well as in the adrenal gland. Androstenedione is synthesized in the adrenals and gonads after activation of steroidogenic enzymes such as 3β-hydroxysteroiddehydrogenase and 17β-hydroxysteroiddehydrogenase and it can also be generated in peripheral tissues from its precursor dehydroepiandrosterone. Besides its mainly ovarian production, testosterone can also be produced by conversion from androstenedione by 17β-hydroxysteroiddehydrogenase type 5 in adipose and other peripheral tissues [10].

The current clinical practice guidelines from the Endocrine Society recommend the use of elevated total, bioavailable, or free serum testosterone level for PCOS diagnosis [11]. However, O'Reilly et al. [12] recently conducted an interesting study demonstrating that high androstenedione levels are a sensitive indicator of PCOS-related androgen excess. The authors further suggested that simultaneous measurement of serum testosterone and androstenedione represents a useful tool for predicting metabolic risk in PCOS. In contrast, results of a previous study indicated that hyperinsulinemia and IR increase ovarian testosterone production in PCOS women but have no effect on androstenedione secretion [13]. It has also been shown that in PCOS women with biochemical hyperandrogenemia, obesity significantly lowers serum androstenedione levels and increases testosterone to androstenedione ratio [14].

Considering those previous inconsistent results on the role of different androgens in the metabolic phenotype of PCOS, we aim to evaluate the association between various androgens including androstenedione, testosterone, and free testosterone and metabolic disturbances in a large cohort including 706 PCOS and 140 BMI-matched control women.

## Methods

### Subjects

The study cohort consisted of 706 women with PCOS, aged 16–45 years, and 140 BMI-matched control women within the same age range. PCOS women were routinely referred to our outpatient clinic for PCOS evaluation from 2007 to 2012. PCOS women consulted our outpatient clinic for PCOS-related symptoms such as hirsutism, acne, obesity, infertility or menstrual irregularities or were referred to our outpatient clinic by gynecologists for further endocrine and metabolic evaluation because of PCO. PCOS was diagnosed using the Rotterdam criteria [15]. Two out of the following three characteristics are required to confirm the diagnosis: clinical and/or biochemical signs of hyperandrogenism, oligo- and/or anovulation, PCO (by ultrasound). Hyperandrogenism was defined by the clinical presence of hirsutism (modified Ferriman-Gallwey score ≥6), acne or alopecia and/or elevated androgen levels (normal range of testosterone: <2.67 nmol/l, free testosterone <11 pmol/l, and free androgen index (FAI): <5.5). Oligo- and/or anovulation were defined by the presence of oligomenorrhea or amenorrhea. PCO morphology was examined by ultrasound [15]. Hyperprolactinemia, Cushing's syndrome, congenital adrenal hyperplasia, and androgen secreting tumors were excluded by specific laboratory analysis (prolactin, cortisol, adrenocorticotropic hormone, and 17α-hydroxyprogesterone).

Healthy women, who were routinely referred to our outpatient clinic for thyroid evaluation between 2009 and 2010, were included in the study as a control group. As thyroid diseases are common in Austria, many women are referred to our outpatient clinic for thyroid evaluation by general practitioners for several reasons including fatigue, obesity, weight gain, a family history of thyroid disease, dysphagia, and many other reasons. All control women had normal thyroid function, regular menstrual cycles, normal serum androgens, and no clinical signs of hyperandrogenism. PCOS and control women did not take any medication known to affect endocrine parameters, carbohydrate metabolism or serum lipid profile for at least 3 months before entering the study.

Data from PCOS as well from control women have been published previously in part [3], [4], [5], [16], [17], [18]. Of note, data on androgen subgroups based on androstenedione and free testosterone as well as androstenedione/free testosterone-ratio are novel and have not been published before.

The study protocol was approved by the ethics committee of the Medical University of Graz (“Ethikkommission der Medizinischen Universität Graz”) and written informed consent was obtained from each patient.

### Procedures

Procedures have been described previously by our group [16]. Standard anthropometric data were obtained from each subject: height, weight, waist and hip circumference, and blood pressure (BP). The basal blood samples for hormone—testosterone, sex hormone binding globulin (SHBG), androstenedione, dehydroepiandrosterone sulphate (DHEAS), thyroid-stimulating hormone, free triiodothyronine, free thyroxine, 17α-hydroxyprogesterone, prolactin, cortisol, and adrenocorticotropic hormone -and metabolic parameters (glucose, insulin, serum lipids) were collected between 8.00 and 9.00 AM after an overnight fast. Assays were performed in the same laboratory as they came in, and the laboratory kits and assays did not change between 2007 and 2012. Both testosterone and SHBG were measured on a daily basis and were stored at 4°C until analysis. Androstenedione and DHEAS levels were measured on a weekly basis, and blood samples were frozen and stored at −40°C until analysis. Free testosterone was calculated from testosterone, SHBG, and albumin according to Vermeulen et al. (16). Elevated free testosterone levels were defined as the upper quartile of free testosterone levels of PCOS women (>11 pmol/l) and elevated androstenedione levels were defined as >11 nmol/l as suggested by the manufacturer and validated in our laboratory. Free testosterone and androstenedione concentrations were used to categorize PCOS women into 4 groups: normal androstenedione and normal free testosterone (NA/NFT), elevated androstenedione and normal free testosterone (HA/NFT), normal androstenedione and elevated free testosterone (NA/HFT), and elevated androstenedione and elevated free testosterone (HA/HFT). We used elevated free testosterone rather than total testosterone levels for categorization as free testosterone is more closely associated with metabolic disturbances in PCOS women (table 1). The FAI was calculated as testosterone (nmol/l)/SHBG (nmol/l)x100. All participants underwent a fasting 75 g oral glucose tolerance test (OGTT). Blood samples were drawn after 30, 60, and 120 minutes for glucose and insulin determination. The area under the glucose response curve and the area under the insulin response curve (AUCins) were calculated according to the trapezoidal method.

**Table 1:** Pearson correlation analyses of androgen levels with clinical and biochemical parameters in PCOS women (n = 706).

	Free testosterone		Testosterone		Androstenedione		Androstenedione/free testosterone-ratio	
*Endocrine characteristics*								
Testosterone	0.735	<0.001	1		0.553	<0.001	−0.175	<0.001
Androstenedione	0.459	<0.001	0.553	<0.001	1		0.444	<0.001
Androstenedione/free testosterone-ratio	−0.518	<0.001	−0.234	<0.001	0.444	<0.001	1	
SHBG	−0.649	<0.001	−0.042	0.298	−0.100	0.014	0.567	<0.001
FAI	0.967	<0.001	0.574	<0.001	0.387	<0.001	−0.560	<0.001
DHEAS	0.286	<0.001	0.222	<0.001	0.350	<0.001	0.094	0.042
*Clinical characteristics*								
Age	−0.138	0.002	−0.136	<0.001	−0.204	<0.001	−0.051	0.265
BMI	0.390	<0.001	0.085	0.037	−0.027	0.510	−0.431	<0.001
Waist circumference	0.342	<0.001	−0.008	0.862	−0.096	0.031	−0.447	<0.001
Waist-to-hip ratio	0.245	<0.001	0.085	0.037	−0.027	0.510	−0.386	<0.001
Total body fat	0.196	0.006	0.035	0.622	0.014	0.854	−0.221	0.003
Fat mass	0.368	<0.001	0.112	0.119	0.039	0.539	−0.404	<0.001
Subcutaneous adipose tissue mass	0.329	<0.001	0.097	0.174	0.030	0.679	−0.370	<0.001
Visceral adipose tissue mass	0.321	<0.001	0.088	0.220	0.042	0.568	−0.329	<0.001
Systolic BP	0.218	<0.001	0.112	0.013	0.065	0.147	−0.171	0.001
Diastolic BP	0.190	<0.001	0.133	0.003	0.075	0.093	−0.143	0.004
Ferriman-Gallwey score	0.213	<0.001	0.044	0.301	−0.011	0.794	−0.191	<0.001
*Metabolic characteristics*								
Fasting glucose	0.148	0.001	0.094	0.021	0.090	0.029	−0.099	0.032
2 h glucose	0.179	<0.001	−0.009	0.838	0.018	0.667	−0.196	<0.001
AUCgluc	0.135	0.015	0.019	0.688	0.033	0.491	−0.148	0.007
HbA1c	0.219	<0.001	0.050	0.237	0.045	0.291	−0.174	<0.001
Fasting insulin	0.340	<0.001	0.088	0.035	0.071	0.087	−0.314	<0.001
Insulin 2 h	0.303	<0.001	0.101	0.017	0.093	0.028	−0.314	<0.001
AUCins	0.283	<0.001	0.088	0.074	0.020	0.688	−0.288	<0.001
HOMA	0.351	<0.001	0.100	0.017	0.078	0.062	−0.314	<0.001
QUICKI	−0.340	<0.001	−0.093	0.027	−0.078	0.062	0.303	<0.001
Matsuda-index	−0.309	<0.001	−0.068	0.171	−0.067	0.183	0.295	<0.001
Oral disposition index	−0.055	0.336	0.039	0.435	−0.075	0.128	0.001	0.991
Triglycerides	0.223	<0.001	0.100	0.014	−0.013	0.746	−0.269	<0.001
Total cholesterol	0.034	0.461	0.113	0.006	0.021	0.608	−0.005	0.919
LDL cholesterol	0.094	0.064	−0.025	0.572	−0.042	0.343	−0.132	0.008
HDL cholesterol	−0.235	<0.001	0.043	0.295	0.076	0.063	0.344	<0.001
Total cholesterol/HDL cholesterol-ratio	0.212	<0.001	0.010	0.812	−0.081	0.049	−0.322	<0.001

PCOS polycystic ovary syndrome, SHBG sex-hormone binding globulin, FAI free androgen index, DHEAS dehydroepiandrosterone sulphate, BMI body mass index, BP blood pressure, AUC area under the curve, HOMA-IR homeostatic model assessment insulin resistance, QUICKI quantitative insulin sensitivity check index, LDL low density lipoprotein, HDL high density lipoprotein.

Obesity was defined as BMI ≥30 kg/m^2^ and overweight as BMI 25–29.9 kg/m^2^. IR was estimated using the homeostatic model assessment-insulin resistance (HOMA-IR) and was assumed for levels >2.5. Quantitative insulin sensitivity check index was used for estimation of insulin sensitivity [19]. We further calculated insulin sensitivity index-Matsuda as an insulin sensitivity index that reflects a composite estimate of hepatic and muscle insulin sensitivity determined from OGTT data (Matsuda-index  = 10,000/√((fasting glucose × fasting insulin) × (glucose_OGTTmean_ × insulin_OGTTmean_))) [20]. It has been shown that Matsuda-index correlates reasonably well with estimates of whole body insulin sensitivity determined by the glucose clamp [21]. We further calculated the oral disposition index as composite measure of β-cell function (Δinsulin_0–30_/Δglucose_0–30_x1/fasting insulin).

Prediabetes and type 2 diabetes mellitus (T2DM) were defined according to the American Diabetes Association [22]. The MS was defined by the National Cholesterol Education Program and the Adult Treatment Panel-III in women presenting at least three of the following criteria: waist circumference >88 cm, high density lipoprotein (HDL) cholesterol<50 mg/dl, triglycerides (TG) level >150 mg/dl, raised BP (systolic >130 mmHg, diastolic >85 mmHg), and raised fasting glucose (>110 mg/dl) or prevalent T2DM.

### Biochemical analyses

Testosterone, insulin, prolactin, thyroid-stimulating hormone, free triiodothyronine, free thyroxine and cortisol (Siemens, Erlangen, Germany) were measured by luminescence immunoassay (intra- and interassay coefficients of variation (CVs) of 6.2% and 4.7% for testosterone, and 4.0% and 2.6% for insulin, respectively). SHBG was measured by luminescence immunoassay (Roche, Basel, Switzerland) with an intra- and interassay CV of 1.3% and 2.1%, respectively. DHEAS (LDN Labor Diagnostika Nord GmbH, Nordhorn, Germany), androstenedione, and 17α-hydroxyprogesterone (DiaMetra, BioVendor, Brno, Czech Republic) were measured by enzyme linked immunosorbent assay with intra- and interassay CV of <10%. Glucose, TG, total cholesterol, HDL cholesterol, and LDL cholesterol were determined using Modular Analytics SWA (Roche). Albumin was measured by photometric assay (Roche, Vienna, Austria).

### Lipometry

Measurements of subcutaneous adipose tissue thickness were performed by means of a patented optical device (EU Patent No. 0516251) on 15 anatomically well-defined body sites as described previously [7]. Lipometry data were available in 196 PCOS and 95 control women.

### Statistical analyses

The control group consists of BMI-matched healthy women. Data are presented as median with interquartile range. The distribution of data was analysed by descriptive statistics and Kolmogorov-Smirnov test. Variables following a non-normal distribution were log transformed and rechecked for normal distribution before being entered in logistic regression analyses. The variables followed normal distribution after log-transformation. ANOVA (with Bonferroni correction), χ^2^-test, and general linear model analyses (age- and BMI-adjusted) were used for comparisons between groups. A power analysis was performed using GPower [23] for all variables independently associated with androgen groups in age- and BMI-adjusted analyses. We performed Pearson correlation analyses to evaluate the correlation of free testosterone, testosterone, androstenedione, and androstenedione/free testosterone-ratio with clinical and biochemical parameters. We performed multiple linear regression analyses with various metabolic parameters as dependent variable and age, BMI, and free testosterone (testosterone, androstenedione, or androstenedione/free testosterone-ratio) as independent variables. We calculated binary logistic regression analyses using IR, prediabetes/T2DM and MS as dependent variables and androgen groups (or androstenedione/free testosterone-ratio quartiles), age, and BMI as independent variables.

All statistical procedures were performed with SPSS version 21 (SPSS Inc., Chicago, IL, USA). A p-value <0.05 was considered statistically significant.

## Results

Characteristics of PCOS and control women are presented in table 2. The prevalence of BMI <25 kg/m^2^, overweight and obesity was 55.7%, 20.1%, and 24.1% in PCOS women and 59.8%, 22.8%, and 17.3% in healthy controls (p = 0.234).

**Table 2:** Clinical and biochemical characteristics of PCOS and control women. PCOS women were stratified according to androgen subgroups.

	Control women		PCOS		NA/NFT		HA/NFT		NA/HFT		HA/HFT	
	(n = 140)		(n = 706)		(n = 354)		(n = 179)		(n = 65)		(n = 108)	
	Median	IQR	Median	IQR	Median	IQR	Median	IQR	Median	IQR	Median	IQR
*Clinical characteristics*												
Age (years)	29	23–31	27	23–31	28	24–33	27^d^	23–29	26^b^^,^ d	22–30	25^b^^,^ d	22–28
BMI (kg/m^2^)	23.6	20.9–28.1	24.2	21.3–30.1	24.2	21.5–29	22.2^d^	20.1–24.4	27.7^b^^,^ d	22.9–33.1	27.4^d^	23.2–34.2
Waist circumference (cm)	81	75–92	82	72–96	83	73–95	74^d^	69–82	91^b^^,^ d	77–108	90^b^	77–102
Waist-to-hip ratio	0.83	0.78–0.87	0.79	0.73–0.87	0.8	0.74–0.87	0.75^b^^,^ d	0.71–0.81	0.84	0.75–0.94	0.82	0.75–0.88
Total body fat (%)	26.5	21.0–30.5	25.6	22.6–29.2	25.3	21.9–29.4	26.5	22.2–29.1	24.8	22.7–28.1	25.6	24.1–29.6
Fat mass (kg)	17.3	12.4–24.3	18.3	13.8–23.4	17.5	13.4–21.0	17.2	12.9–20.3	25.2^d^	22.5–27.2	21.5^d^	16.7–26.7
Subcutaneous adipose tissue mass (kg)	14.2	10.1–19.2	15.9	12.1–19.0	15.0	11.3–17.9	15.5	11.1–18.8	18.9	17.5–20.9	18.0	14.5–21.8
Visceral adipose tissue mass (kg)	3.1	2.2–4.7	2.5	1.6–4.0	2.4	1.5–3.3	2.0	1.4–2.9	5.9^d^	3.1–7.1	3.1^d^	2.3–5.8
Systolic BP (mmHg)	116	110–124	118	110–130	115	107–127	116	110–126	124^b^	113–135	124	112–132
Diastolic BP (mmHg)	76	68–83	80^b^	74–89	79^b^	70–86	80	73–85	83^b^	75–90	85^b^^,^ d	75–91
Ferriman-Gallwey score	2	0–4	6^b^	2.0–11.0	7^b^	3–11	5	1–9	7^b^	3–12	7^b^	3–12
*Metabolic characteristics*												
Fasting glucose (mmol/l)	4.7	4.4–5.1	4.7	4.4–5.0	4.7	4.3–5	4.7	4.4–5	4.7	4.4–5.1	4.7^d^	4.4–5.2
2 h glucose (mmol/l)	5.2	4.3–5.9	5.3	4.5–6.4	5.3	4.5–6.5	5.1	4.3–6.2	5.9^d^	5.3–7.5	5.7^d^	5.0–7.3
AUCgluc	160.9	138.8–189.8	172.3^a^	151.3–198.8	174	149.3–196	168.9	149.1–189	179.3^a^^,^ c	167–198.8	174.8^c^	153.3–212.3
HbA1c (%)	5.2	5–5.5	5.2	5–5.3	5.1^d^	5.0–5.3	5.1^d^	4.9–5.2	5.3^d^	5.1–5.8	5.2^d^	5.0–5.4
Fasting insulin (µU/ml)	4.7	2.5–7.5	6.2^a^	3.8–9.8	6^b^	3.9–8.9	5	3.1–8	8.7^b^^,^ d	5–13.8	9.1^b^^,^ d	5.4–14.3
Insulin 2 h (µU/ml)	27.9	16.2–47.7	33.4^a^	20–58	31.5	19.0–50.2	30	16.5–50.1	55.9^b^^,^ d	28.5–87.8	45^b^^,^ d	25.4–81.1
AUCins	53.9	36.5–78.3	61.6^a^	39.4–96.7	57.4	37.7–80.3	49.2	31.5–73.8	88.4^b^^,^ d	59.7–124.5	77.3^b^^,^ d	52.6–132.4
HOMA-IR	1.0	0.5–1.6	1.3^b^	0.8–2.2	1.2^b^	0.8–1.9	1.1	0.6–1.8	1.8^b^^,^ d	1.1–3	2.0^b^^,^ d	1.1–3.1
QUICKI	0.38	0.35–0.43	0.37^a^	0.34–0.4	0.37	0.35–0.4	0.38	0.35–0.41	0.35^b^^,^ d	0.32–0.38	0.34^b^^,^ d	0.32–0.38
Matsuda-index	8.8	5.0–15.7	7.3^a^	4.5–11.30	7.4	2.0–11.5	8.6	5.6–13.5	4.8^b^^,^ d	2.8–8.3	4.9^b^^,^ d	3.2–7.9
Oral disposition index	3.3	1.8–7.0	2.5	1.5–4.4	2.5	2.5–4.3	2.5	1.4–4.8	2.5	1.8–4.8	1.9^b^	1.2–3.8
Triglycerides (mmol/l)	0.8	0.6–1.2	0.9	0.6–1.2	0.8^d^	0.6–1.1	0.7^d^	0.6–1.0	1.1^d^	0.8–1.5	1.1^d^	0.8–1.4
Total cholesterol (mmol/l)	4.8	4.2–5.4	4.6^a^	4.0–5.2	4.6	4.1–5.2	4.7	4–5.1	4.7	4.0–5	4.7	4.1–5.3
LDL cholesterol (mmol/l)	2.6	2.2–33	2.6	2.2–3.1	2.6	2.2–3.1	2.5	2–2.9	2.6	2–3.1	2.6	2.2–3.1
HDL cholesterol (mmol/l)	1.9	1.6–2.2	1.7^b^	1.4–2.0	1.7^b^	1.3–2	1.8^d^	1.6–2.1	1.5^b^	1.3–1.9	1.6^b^	1.3–1.9
TC/HDL cholesterol-ratio	2.6	2.3–3.3	2.7	2.3–3.4	2.8	2.3–3.4	2.5^d^	2.1–3.1	2.8^d^	2.2–3.7	3.1^d^	2.5–3.9
IR (%)	7.9		22.7^b^		15.4^a^		12.3		44.2^b^^,^ d		48.5^b^^,^ d	
Prediabetes/T2DM (%)	13.4		13.8		12.2		9.6		33.3^d^		15.5	
MS (%)	7.7		11.8		9.4		4.3		17.9^b^^,^ c		29.1^b^^,^ c	
*Endocrine characteristics*												
Testosterone (nmol/l)	1.5	1.1–1.9	2.2^b^	1.7–2.8	1.7^b^	1.4–2.1	2.4^b^^,^ d	2.0–2.8	2.8^b^^,^ d	2.4–3.2	3.2^b^^,^ d	2.7–3.9
Free testosterone (pmol/l)	4.0	3.0–7.0	9.0^b^	7.0–13.0	7.0^b^	5.0–9.0	9.0^b^^,^ d	7.0–11.0	17.5^b^^,^ d	16.0–19.5	17.0^b^^,^ d	16.0–19.0
Androstenedione/free testosterone-ratio	1.35	0.94–2.35	1.05^b^	0.75–1.47	0.95^b^	0.72–1.25	1.71^d^	1.26–2.42	0.58 b ^,^ d	0.48–0.77	1.13^d^	0.83–1.42
FAI	2.0	1.1–3.2	5.0^b^	3.2.7.7	3.7^b^	2.4–5.2	4.6^b^	3.4–5.8	11.3^b^^,^ d	9.5–14.3	10.9^,^ d	8.1–14.2
SHBG (nmol/l)	70.8	49.6–111.8	45.4^b^	29.8–63.0	48.5^b^	32.6–66.3	53.5^b^	37.1–73.6	30.6^b^^,^ d	22.5–41.2	35.0^b^^,^ d	23.5–48.4
Androstenedione (nmol/l)	5.4	4.2–8.7	10.1^b^	7.0–14.8	7.0^b^	5.5–8.7	13.9^b^^,^ d	11.9–18.7	8.8^b^^,^ d	7.4–9.5	16.6^b^^,^ d	14.0–22.0
DHEAS (µmol/l)	2.5	1.8–3.8	5.2^b^	3.6–7.2	4.4^b^	3.0–6.0	5.5^b^	3.8–7.4	5.6^b^	4–7.5	6.7^b^^,^ d	5.1–9.1

a p<0.01.

b p<0.001 compared to healthy controls.

c p<0.01.

d p<0.001 compared to NA/NT group.

Comparisons between groups were performed using ANOVA or χ^2^-test.

PCOS polycystic ovary syndrome, NA normal androstenedione, NFT normal free testosterone, HA high androstenedione, HFT high free testosterone, IQR interquartile range, BMI body mass index, BP blood pressure, AUC area under the curve, HOMA-IR homeostatic model assessment insulin resistance, QUICKI quantitative insulin sensitivity check index, LDL low density lipoprotein, HDL high density lipoprotein, T2DM type 2 diabetes mellitus, MS metabolic syndrome, FAI free androgen index, SHBG sex-hormone binding globulin, DHEAS dehydroepiandrosterone sulphate.

Hyperandrogenism was present in 85.6%, menstrual irregularities in 85.5%, and PCO in 56.8% of PCOS women, respectively. We found hyperandrogenism and menstrual irregularities and PCO in 50.6%, hyperandrogenism and menstrual irregularities in 32.9%, hyperandrogenism and PCO in 6.8%; and menstrual irregularities and PCO in 9.8% of PCOS women, respectively.

Correlation analyses of androgens with clinical and biochemical variables are shown in table 1. In linear regression analyses, the association of free testosterone with fasting and 2 h insulin, Matsuda-index, HbA1c, and HDL cholesterol remained significant whereas the association with other metabolic parameters lost significance. Moreover, the association between testosterone and diastolic BP and total cholesterol remained significant whereas the other associations lost significance in multiple linear regression analyses. When using androstenedione as independent variable in multiple linear regression analyses, all associations (except Ferriman-Gallwey score) lost significance.

### Androgen groups

Clinical and biochemical characteristics of PCOS women according to androgen groups are shown in table 2. In age- and BMI-adjusted models, the association between androgen groups and glucose 2 h (p = 0.049), HbA1c (p = 0.003), fasting insulin (p = 0.029), insulin 2 h (p = 0.029), AUCins (p = 0.015), HOMA-IR (p = 0.004), Matsuda-index (p = 0.004), TG (p = 0.010), total cholesterol (p = 0.001), and HDL cholesterol (p = 0.015) remained significant whereas the other associations between androgen groups and metabolic parameters lost significance.

Power analyses revealed a power of >99% to detect a statistical significant difference for all independently associated variables except total cholesterol (power 87.7%).

### IR, prediabetes/T2DM, and MetS

**Figure 1:**
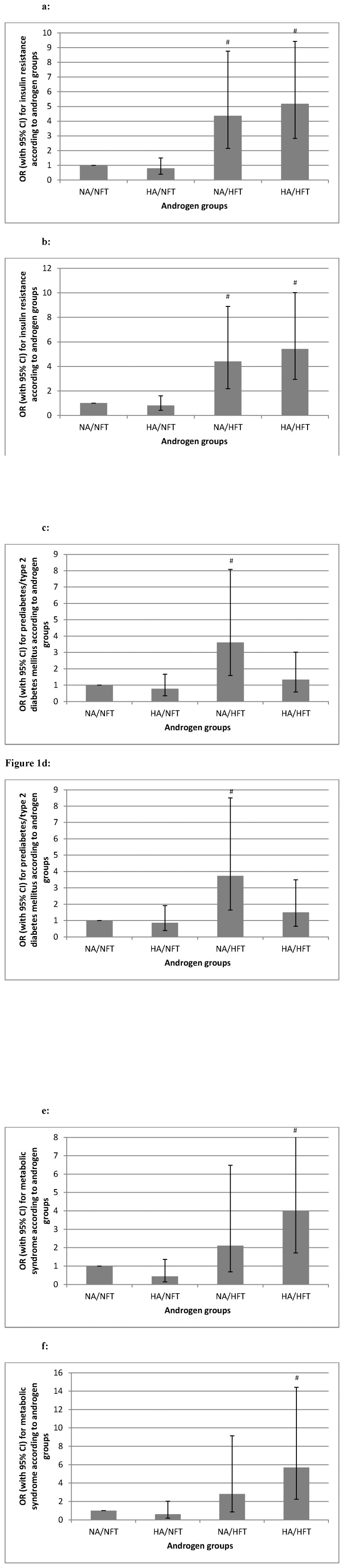
Risk of metabolic disturbances according to androgen groups. OR with 95% CI for insulin resistance, prediabetes/type 2 diabetes mellitus, and metabolic syndrome according to androgen groups (NA/NFT n = 354, HA/NFT n = 179, NA/HFT n = 65, HA/HFT n = 108). # p-value <0.05. Figure 1a: Binary logistic regression analysis using insulin resistance as dependent variable and androgen groups as independent variable (p<0.001 NA/HFT vs NA/NFT, p<0.001 HA/HFT vs NA/NFT). Figure 1b: Binary logistic regression analysis using insulin resistance as dependent variable and age and androgen groups as independent variables (p<0.001 NA/HFT vs NA/NFT, p<0.001 HA/HFT vs NA/NFT). Figure 1c: Binary logistic regression analysis using prediabetes/type 2 diabetes mellitus as dependent variable and androgen groups as independent variable (p = 0.002 NA/HFT vs NA/NFT). Figure 1d: Binary logistic regression analysis using prediabetes/type 2 diabetes mellitus as dependent variable and age and androgen groups as independent variables (p = 0.002 NA/HFT vs NA/NFT). Figure 1e: Binary logistic regression analysis using metabolic syndrome as dependent variable and androgen groups as independent variable (p<0.001 HA/HFT vs NA/NFT). Figure 1f: Binary logistic regression analysis using metabolic syndrome as dependent variable and age and androgen groups as independent variables (p<0.001 HA/HFT vs NA/NFT).

Binary logistic regression analyses using IR, prediabetes/T2DM and MS as dependent variables are shown in figure 1. The association of IR with HA/HFT remained stable (p = 0.007) but was attenuated for NA/HFT after additional adjustment for BMI. Results were attenuated after additional adjustment for BMI for prediabetes/T2DM (p = 0.067) and MS (p>0.100).

### Androstenedione/free testosterone-ratio

**Figure 2:**
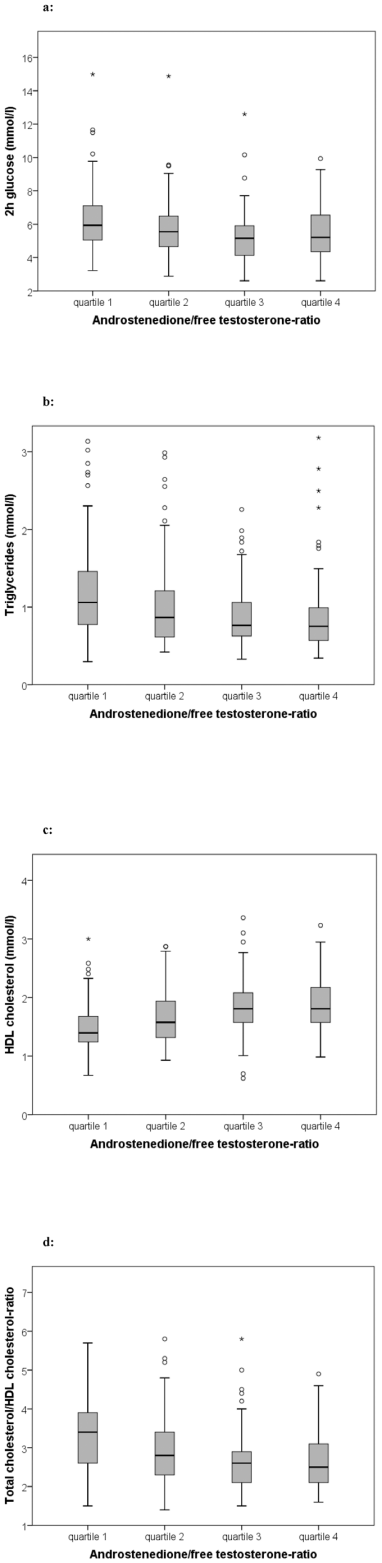
Metabolic parameters according to androstenedione/free testosterone quartiles. Metabolic parameters according to androstenedione/free testosterone-ratio quartiles in PCOS women. ANOVA and general linear model adjusted for age and BMI were used for comparison between groups. (quartile 1 n = 176, quartile 2 n = 177, quartile 3 n = 177, quartile 4 n = 176). Figure 2a: 2 h glucose levels in PCOS women according to androstenedione/free testosterone-ratio quartiles (crude: p<0.001, age and BMI-adjusted p = 0.030). Figure 2b: Triglyceride levels in PCOS women according to androstenedione/free testosterone-ratio quartiles (crude: p<0.001, age and BMI-adjusted p = 0.040). Figure 2c: HDL cholesterol levels in PCOS women according to androstenedione/free testosterone-ratio quartiles (crude: p<0.001, age and BMI-adjusted p = 0.001). Figure 2d: Total cholesterol/HDL cholesterol-ratio in PCOS women according to androstenedione/free testosterone-ratio quartiles (crude: p<0.001, age and BMI-adjusted p = 0.005).

**Figure 3:**
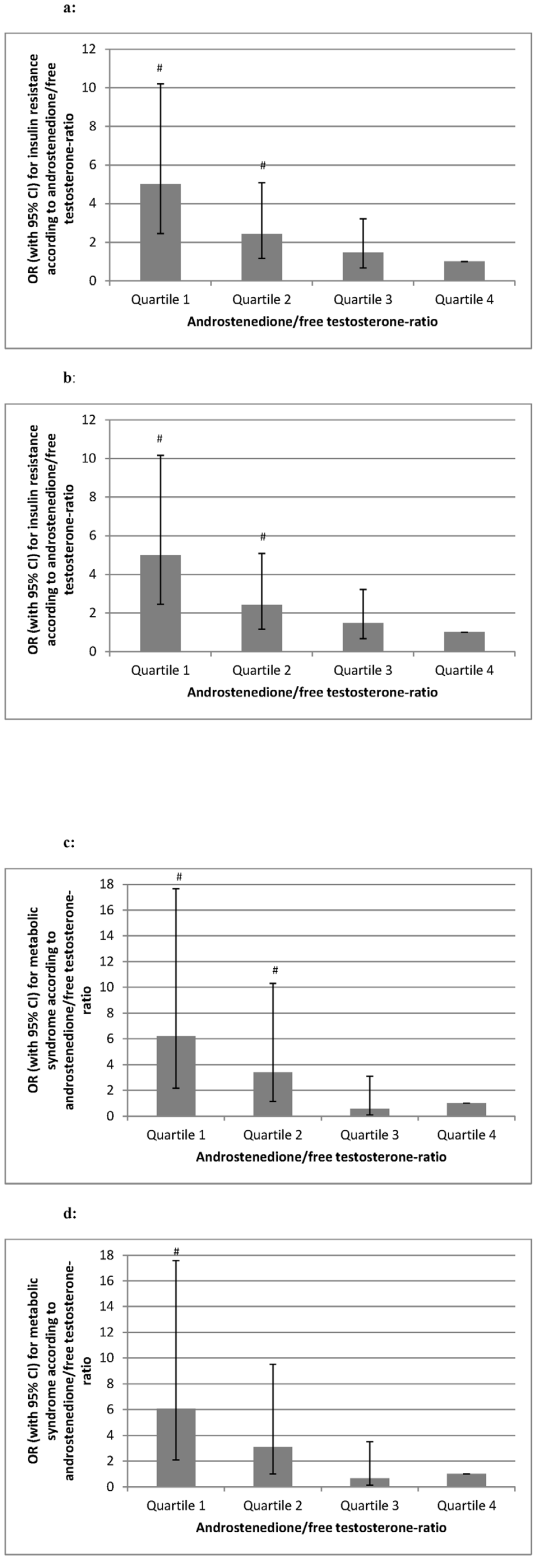
Risk of metabolic disturbances according to androstenedione/free testosterone quartiles. OR with 95% CI for insulin resistance and metabolic syndrome according to androstenedione/free testosterone-ratio quartiles (quartile 1 n = 176, quartile 2 n = 177, quartile 3 n = 177, quartile 4 n = 176). # p-value <0.05. Figure 3a: Binary logistic regression analysis using insulin resistance as dependent variable and androstenedione/free testosterone-ratio quartiles as independent variable (p = 0.019 quartile 2 vs. quartile 4, p<0.001 quartile 1 vs quartile 4). Figure 3b: Binary logistic regression analysis using insulin resistance as dependent variable and age and androstenedione/free testosterone-ratio quartiles as independent variables (p = 0.019 quartile 2 vs. quartile 4, p<0.001 quartile 1 vs quartile 4). Figure 3c: Binary logistic regression analysis using metabolic syndrome as dependent variable and androstenedione/free testosterone-ratio quartiles as independent variable (p = 0.030 quartile 2 vs. quartile 4, p = 0.001 quartile 1 vs quartile 4). Figure 3d: Binary logistic regression analysis using metabolic syndrome as dependent variable and age and androstenedione/free testosterone-ratio quartiles as independent variables (p = 0.050 quartile 2 vs. quartile 4, p = 0.001 quartile 1 vs quartile 4).

Characteristics of PCOS women according to androstenedione/free testosterone-ratio quartiles are shown in table 3 and figure 2, correlation analyses of androstenedione/free testosterone-ratio are shown in table 1. In linear regression analyses, the association of androstenedione/free testosterone-ratio with AUCins, HOMA-IR, Matsuda-index, HDL cholesterol, and total cholesterol/HDL cholesterol-ratio remained significant and was attenuated for fasting insulin (p = 0.059), 2 h insulin (p = 0.059), and TG (p = 0.058) whereas the association with other metabolic parameters lost significance. Binary logistic regression analyses using IR and MS as dependent variable are shown in figure 3. Results were attenuated after additional adjustment for BMI (p>0.100). We found no significant association of androstenedione/free testosterone-ratio quartiles with prediabetes/T2DM in multivariate analyses (data not shown).

**Table 3:** Clinical and biochemical characteristics of PCOS women according to androstenedione/free testosterone-ratio quartiles.

	Androstenedione/free testosterone-ratio quartiles							
	quartile 1 (<0.75)		quartile 2 (0.75–1.05)		quartile 3 (1.06–1.47)		quartile 4 (>1.47)	
	(n = 176)		(n = 177)		(n = 177)		(n = 176)	
	Median	IQR	Median	IQR	Median	IQR	Median	IQR
*Clinical characteristics*								
Age (years)	27	23–33	27	24–31	26	23–31	27	23–30
BMI (kg/m^2^)	27.9	24–35.3	25.5^a^	23.1–30.4	22.3^b^^,^ d	20.5–27.0	21.6^c^^,^ e	20–30
Waist circumference (cm)	94	82–112	85^a^	79–96	80^b^^,^ d	72–88	73^c^^,^ e	69–82
Waist-to-hip ratio	0.85	0.78–0.95	0.81	0.76–0.89	0.78^b^^,^ d	0.74–0.84	0.75^c^^,^ e	0.71–0.81
Total body fat (%)	25.8	23.6–28.8	26.8	24.3–30.4	23.4^b^^,^ d	19.8–26.1	23.9^e^	17.0–29.1
Fat mass (kg)	21.8	17.3–26.5	19.6	16.1–23.3	15.0^b^^,^ d	11.4–18.8	13.8^c^^,^ e	10.7–19.6
Subcutaneous adipose tissue mass (kg)	17.4	14.5–20.8	16.5	13.9–19.3	12.6^b^^,^ d	10.1–17.1	12.4^c^^,^ e	8.0–17.4
Visceral adipose tissue mass (kg)	3.5	2.2–5.9	2.7	1.8–4.0	1.8^b^	1.4–2.8	1.9^c^	1.3–2.7
Systolic BP (mmHg)	122	110–139	120	110–128	114^b^	105–125	115^c^	109–125
Diastolic BP (mmHg)	85	74–96	80	74–87	76^b^	70–84	80^c^	72–87
Ferriman-Gallwey score	9	4.0–13	7	3.0–11.0	5 b	2.0–9.0	5	2.0–10.0
*Metabolic characteristics*								
Fasting glucose (mmol/l)	4.8	4.5–5.2	4.7	4.3–5.1	4.6^b^	4.3–5.0	4.7^c^	4.4–5.0
2 h glucose (mmol/l)	5.9	5.1–7.1	5.6	4.7–6.5	5.2^b^^,^ d	4.1–5.9	5.2^c^	4.4–6.5
AUCgluc	186.6	159.5–211.8	176.6	151–208.8	168.8^b^	148.8–192.5	169^c^	151.8–194.8
HbA1c (%)	5.2	5–5.4	5.1^a^	5.0–5.3	5.1^b^	5.0–5.3	5.1^c^	4.9–5.3
Fasting insulin (µU/ml)	8.6	5.9–14.3	6.7	4.3–10.5	5.2^b^	3.1–8.5	5^c^^,^ e	3.0–7.5
Insulin 2 h (µU/ml)	47.4	23.5–91.3	39.6	23–59.5	26.4^b^	17.8–48.8	28.7^c^^,^ e	18.8–45.1
AUCins	80.2	50.2–124.5	70.4	54.8–92.4	53.1^b^	35.9–75.6	48.4^c^^,^ e	35.2–74.7
HOMA-IR	1.9	1.2–3.4	1.3^a^	0.9–2.3	1.0^b^^,^ d	0.6–1.8	1.1^c^^,^ e	0.6–1.6
QUICKI	0.35	0.32–0.37	0.37	0.34–0.39	0.38^b^	0.35–0.42	0.38^c^	0.35–0.41
MATSUDA-index	4.9	2.6–8.5	6.2	4.1–9	8.7^b^	5.9–11.6	8.6^c^^,^ e	5.6–12.9
Oral disposition index	2.4	1.4–4.4	2.7	1.5–5.1	2.8	1.7–6.1	2.4	1.4–4.2
Triglycerides (mmol/l)	1.1	0.8–1.5	0.9^a^	0.6–1.2	0.8^b^	0.6–11	0.8^c^	0.6–1
Total cholesterol (mmol/l)	4.6	4.0–5.2	4.6	4–5.1	4.5	4–5.2	4.6	4.1–5.1
LDL cholesterol (mmol/l)	2.6	2.4–3.3	2.6	2.1–2.9	2.4	2.1–3	2.4	2.1–2.9
HDL cholesterol (mmol/l)	1.4	1.2–1.7	1.6^a^	1.3–1.9	1.8 b ^,^ d	1.6–2.1	1.8^c^^,^ e	1.6–2.2
TC/HDL cholesterol-ratio	3.4	2.6–3.9	2.8^a^	2.3–3.4	2.6^b^^,^ d	2.1–2.9	2.5^c^^,^ e	2.1–3.1
IR (%)	38.7		23.5^a^		15.7^b^		11.2^c^^,^ e	
Prediabetes/T2DM (%)	24.2		13.7		4.0^b^^,^ d		14.6^f^	
MS (%)	25.1		15.5		3.0^b^^,^ d		5.1^c^^,^ e	
*Endocrine characteristics*								
Testosterone (nmol/l)	2.3	1.9–3.1	2.1^a^	1.7–2.7	2.2^b^	1.6–2.8	1.9^c^	1.4–2.6
Free testosterone (pmol/l)	14	10.0–18	10^a^	7.0–13.0	9^b^	6.5–11.0	6.0^c^^,^ e ^,^ f	4.0–9
FAI	8.7	5.6–12.7	5.3^a^	3.7–7.6	4.3^b^^,^ d	3.2–6	2.9^c^^,^ e ^,^ f	1.8–4.6
SHBG (nmol/l)	27.5	20.6–40	43.1^a^	30.3–54.9	52.5^b^^,^ d	37.8–65.4	63.2^c^^,^ e ^,^ f	46.3–92.5
Androstenedione (nmol/l)	7.7	5.8–9.7	9^a^	6.5–11.9	10.9^b^^,^ d	8–14.5	13.4^c^^,^ e ^,^ f	9.5–19.8
DHEAS (µmol/l)	5	3.5–6.6	5.5	3.4–7.2	5.2	4–7.4	5.4	4.0–7.4

a p<0.01 quartile 1 vs quartile 2.

b p<0.01 quartile 1 vs. quartile 3.

c p<0.01 quartile 1 vs quartile 4.

d p<0.01 quartile 2 vs. quartile 3.

e p<0.01 quartile 2 vs. quartile 4.

f p<0.01 quartile 3 vs. quartile 4.

Comparisons between groups were performed using ANOVA or χ^2^-test.

PCOS polycystic ovary syndrome, IQR interquartile range, BMI body mass index, BP blood pressure, AUC area under the curve, HOMA-IR homeostatic model assessment insulin resistance, QUICKI quantitative insulin sensitivity check index, LDL low density lipoprotein, HDL high density lipoprotein, T2DM type 2 diabetes mellitus, MS metabolic syndrome, FAI free androgen index, SHBG sex-hormone binding globulin, DHEAS dehydroepiandrosterone sulphate.

## Discussion

This is the first study demonstrating that a high androstenedione/free testosterone-ratio is associated with a beneficial metabolic profile in PCOS women. Further, PCOS women with increased free testosterone levels are at increased metabolic risk whereas PCOS women with isolated androstenedione elevation have a similar risk as PCOS women without hyperandrogenemia. Our results are to some extent in contrast to a recent study showing that PCOS women with elevated androstenedione levels have an adverse metabolic phenotype [12]. While the study has several strengths including the measurement of androgens by tandem mass spectrometry, one limitation is the fact that due to the small sample size (n = 86) all women with elevated testosterone also had elevated androstenedione. Therefore, the authors were not able to analyze a subgroup of PCOS women with normal androstenedione and elevated testosterone (NA/HT). Further, mean BMI was 32 kg/m^2^ in those PCOS women and thus much higher compared to our cohort.

Our results suggest that PCOS women with elevated free testosterone have an adverse metabolic profile compared to PCOS women with NA/NFT or HA/NFT. This might either be related to an adverse effect of free testosterone (which has been consistently shown in PCOS women) but might also be attributed to some beneficial effect of androstenedione. This hypothesis is supported by our interesting results showing an independent association of androstenedione/free testosterone-ratio with hyperinsulinemia, IR, and insulin sensitivity. Of note, BMI-matched control women who are at low metabolic risk have significantly higher androstenedione/free testosterone-ratios than PCOS women despite lower androstenedione and free testosterone levels. Different effects of various androgens on the metabolic phenotype have been proposed previously. It has been suggested that PCOS women with IR will not develop adrenal hyperandrogenism and at the same time will have lower levels of androstenedione, suggesting insulin inhibition of the putative factor responsible for stimulating both adrenal and ovarian androgen production via CYP17 [24]. This notion is supported by the fact that androstenedione and DHEAS are decreased in insulin resistant obese women despite testosterone and FAI remaining unchanged. Interestingly, insulin infusion has been shown to lower androstenedione levels without an effect on testosterone or DHEAS levels [25]. Further, insulin might enhance peripheral steroidogenesis, while inducing a relative impairment of CYP17 activity [26] leading mainly to testosterone excess without androstenedione and DHEAS excess. Thus, hyperinsulinemia and IR increase ovarian testosterone production in PCOS women but have no effect on androstenedione secretion [13]. Likewise, no correlation was found between adrenal androgen secretion (androstenedione response to synthetic corticotropin) and IR in PCOS or eumenorrhoic women [27]. Further, while there is a positive association of testosterone and free testosterone with obesity, serum androstenedione has not been shown to increase with obesity or insulin levels in women with and without hirsutism [28]. Similarly, free testosterone serum concentrations were higher in abdominally obese women than in both the controls and women with lower-body obesity, whereas androstenedione, DHEAS, and estradiol did not differ significantly between the three groups [29]. This is in line with the lack of a significant correlation of androstenedione but a strong correlation of free testosterone with BMI and body fat in our study.

As androstenedione is also produced in significant quantities by the adrenal gland, it is worth mentioning that ovarian and adrenal androgens might have opposing effects on body weight and insulin metabolism in women with PCOS [30], [31], [32]. Some of us demonstrated in a large cohort of PCOS women that women with adrenal hyperandrogenism as indicated by elevated DHEAS have a more beneficial metabolic profile compared to women with ovarian hyperandrogenism defined by elevated free testosterone [16]. A high DHEAS/free testosterone or DHEAS/total testosterone-ratio is associated with a favorable metabolic phenotype in PCOS women [16], [33].

Interestingly, we observed an independent association of androgen groups as well as of androstenedione/free testosterone-ratio quartiles with serum lipids suggesting a beneficial effect of androstenedione on serum lipids but also a negative impact of free testosterone on dyslipidemia. Likewise, a study among adolescent girls with PCOS reported a negative association of androstenedione with TG and a positive association of androstenedione with HDL cholesterol levels [34] whereas testosterone was negatively associated with HDL cholesterol levels. As dyslipidemia is considered an important cardiovascular risk factor in PCOS women [2], this observation is of interest. The origin of dyslipidemia in PCOS is not entirely clear but has been attributed to the effects of hyperandrogenism and IR combined with genetic and environmental factors such as diet and physical activity [18], [35], [36]. Of note, our results suggest that hyperandrogenism is an important factor that should be considered when investigating dyslipidemia in PCOS as women with HA/NFT and with a high androstenedione/free testosterone-ratio present with a favorable lipid profile. In contrast, women with increased free testosterone and low androstenedione/free testosterone -ratio have an adverse lipid profile independent of BMI.

We found few significant differences regarding the metabolic phenotype of PCOS women with NA/NFT and HA/NFT compared to healthy BMI-matched control women. This is in line with a previous studies suggesting that PCOS women without hyperandrogenemia have a metabolic risk that is comparable with healthy BMI-matched women [37]. Nevertheless, the prevalence of IR was higher in PCOS women with NA/NFT and HA/NFT compared to controls indicating that PCOS women without elevated free testosterone levels are also affected by metabolic risk but to a lesser extent. Although androstenedione and free testosterone levels were lower in control women, we found higher androstenedione/free testosterone-ratio which further supports the hypothesis of a potential protective role of high androstenedione/free testosterone-ratio. Altogether, our results clearly indicate that PCOS women with elevated free testosterone levels and a low androstenedione/free testosterone -ratio constitute a high-risk group who are in need of a close metabolic follow-up and intensive treatment, including lifestyle intervention.

We aimed to study the association of various androgens with metabolic disturbances in PCOS but we did not investigate the relationship between androstenedione and free testosterone elevation or its ratio with infertility. It should be underlined that infertility is a major aspect in PCOS and a recent study by Legro et al. [38] demonstrated that there are effective strategies such as letrozole treatment that improve ovulation as well as live birth rates in women affected by PCOS. As PCOS women within the various androgen subgroups differ regarding their metabolic phenotype and metabolic disturbances are also associated with reduced fertility [39], future studies should also focus on the association of various androgens with risk of infertility as well as on a possible impact of different androgens on infertility management.

One limitation of our study is the observational character that does not allow final conclusions as to a potential causal role of various androgens in the development of metabolic disturbances. Second, we did not measure androgen levels with mass spectrometry, which is considered gold standard when measuring androgen concentrations in women [40]. However, given the case that measurements of androgen levels are inaccurate in our cohort, our results would under- rather than overestimate the observed association between androgen concentrations and metabolic disturbances. Further, lipometry data were only available in a subgroup of patients and controls. The strengths of our study are the large sample size of PCOS women as well as the detailed metabolic characterization including glucose and insulin levels derived from oral glucose tolerance tests. We were therefore able to analyze all relevant subgroups of PCOS women.

We present evidence that PCOS women with elevated free testosterone levels have an adverse metabolic profile compared to PCOS women with normal free testosterone levels. Further, higher androstenedione/free testosterone-ratio was independently associated with a beneficial metabolic profile compared to PCOS women with lower androstenedione/free testosterone-ratio. Thus, our study supports the current concept suggesting an independent negative impact of free testosterone on the metabolic phenotype of PCOS women. However, androstenedione levels were not found to have a negative impact on metabolic phenotype but might have some beneficial effect as indicated by the inverse association of androstenedione/free testosterone-ratio with metabolic disturbances and the higher androstenedione/free testosterone-ratio observed in BMI-matched control women. Further studies including a large sample size of PCOS and BMI-matched control women using mass spectrometry are necessary to elucidate the role of various androgens in the metabolic phenotype of PCOS women.
